# Investigation of cross-reactivity between phenobarbital and levetiracetam in children with epilepsy: A prospective, observational multicenter study

**DOI:** 10.1016/j.ebr.2023.100583

**Published:** 2023-01-11

**Authors:** Samane Rahimi, Bardia Danaei, Mohammad Mehdi Nasehi, Sasan Saket, Nazanin Farahbakhsh, Maryam Rajabnejad, Mohammad Mehdi Taghdiri

**Affiliations:** a Department of Pediatric Emergency Medicine, School of Medicine, Mofid Children's Hospital, Shahid Beheshti University of Medical Sciences, Tehran, Iran; b Department of Microbiology, School of Medicine, Shahid Beheshti University of Medical Sciences, Tehran, Iran; c Department of Pediatric Neurology, School of Medicine, Mofid Children's Hospital, Shahid Beheshti University of Medical Sciences, Tehran, Iran; d Pediatric Neurology Research Center, Shahid Beheshti University of Medical Sciences, Tehran, Iran; e Pediatric Pulmonology Department, School of Medicine, Mofid Children's Hospital, Shahid Beheshti University of Medical Sciences, Tehran, Iran; f Pediatric Infections Research Center (PIRC), Research Institute for Children's Health (RICH), Shahid Beheshti University of Medical Sciences, Tehran, Iran

**Keywords:** Drug allergy, Phenobarbital, Levetiracetam, Child neurology, Epilepsy

## Abstract

**Objectives:**

Pharmaceutical allergic reactions due to antiseizure medications (ASMs) are one of the major concerns in the management of patients. Finding an alternative ASM which does not cause allergic reactions and has acceptable effectiveness can be difficult. In this regard, the present study attempts to investigate the cross-reactivity between phenobarbital and levetiracetam in children under treatment for seizure control.

**Materials & Methods:**

The present study is a prospective, observational independent assessor study. 30 children with epilepsy who were hypersensitive to phenobarbital therapy were studied. In order to evaluate the cross-reactivity of the drugs, levetiracetam replaced phenobarbital to control seizure. Within 6 months, any allergic reactions and seizure recurrences were evaluated in the patients.

**Results:**

53 % of the children in this study were female. The mean age of patients was 42.4 months. In patients’ follow up no cross-reactive responses were observed in any of the patients. Seizure recurrence rate was 30 % in the first six months of follow up that with increasing dosage in the second six months of follow-up, decreased to 10 %.

**Conclusion:**

Based on the results of this study, in children with epilepsy controlled by phenobarbital if allergic reactions to phenobarbital occur, levetiracetam may be used as a suitable alternative medicine.

## Highlights

• Anti-epileptic drugs can cause hypersensitivity and immunologic reactions in patients.
• Anti-epileptic drugs cross-reactions can cause hypersensitivity to replaced new drugs.
• Drug cross-reaction and efficacy need to be considered together in seizure management.
• Levetiracetam may be a good choice to replace phenobarbital in drug allergic reaction.

## Introduction

Adverse drug reactions have always been a major concern in treatment. These reactions are undesired responses to a drug that occur with usual therapeutic doses. An allergic drug reaction is one involving the immune system and is caused by immunologic amplification. Although fewer than 15 % of all adverse drug reactions are allergic in nature, they can be very troublesome for the patients and managing them can be challenging [1]. With the introduction of phenobarbital, the modern treatment of epilepsy started in 1912 and flourished since then. Today, more than 30 drugs are included in the pharmacological armamentarium against this disease [2].

According to studies, various degrees of skin manifestations of allergic reactions due to antiseizure medications (ASMs) have been reported. The most common form is mild skin rash and the more serious forms can be described as Steven-Johnson syndrome (SJS), toxic epidermal necrolysis (TEN), and drug reaction with eosinophilia and systemic symptoms (DRESS) [[3], [4]]. In the 5 to 15 % of patients treated with ASMs such as phenytoin, carbamazepine and phenobarbital, the incidence of relatively benign rash has been reported. There are also reports of skin rash following the use of new ASMs, such as oxcarbazepine and lamotrigine [4]. Hypersensitivity to the anticonvulsants is one of the serious allergic reactions which can be caused by these drugs during treatment. The terms, anticonvulsant hypersensitivity syndrome (AHS) or anticonvulsant/drug-induced hypersensitivity syndrome (A/DiHS) are used to refer to such reactions including a triad of symptoms consisting of dermatologic rashes, fever, and evidence of systemic organ involvement. The diagnosis of AHS is mostly based on this triad and clinical judgment [5].

Cutaneous allergic reactions and AHS have been reported in non-aromatic drugs like levetiracetam, valproic acid, topiramate, and etc. but with lesser extent in comparison to aromatic ones such as phenobarbital, phenytoin, and carbamazepine which are well known to create skin eruptions as well as severe AHS [[6], [7]].

Cross-reactivity between drugs is clinically manifested when a new drug administered shows hypersensitivity reactions because of a common pharmacological characteristic or as a consequence of a preexisting sensitization to a structurally similar compound [8]. The importance of considering this phenomenon in clinical setting is that in case of an allergic reaction to ASMs, the wrong choice in selecting alternative ASMs may lead to dangerous reactions such as SJS.

Cross-reactivity has been described among aromatic ASMs. The fact that these drugs are metabolized into reactive intermediates (i.e., toxic arene oxides) can explain the high 75 % rate of cross-reactivity in patients using them [6]. This phenomenon has been reported between aromatic and non-aromatic ASMs but happened rarely in comparison to cross-sensitivity among aromatic ASMs which makes non-aromatic ASMs a rational and preferable choice as a second line treatment if significant allergic reactions occur due to using aromatic ASMs [[4], [9]].

Phenobarbital is one of the most widely available and inexpensive ASMs and the first choice in controlling of almost all types of seizures including childhood seizures in developing countries where the overwhelming priority is to retain patients in treatment through a rationally long period of time. Thus it is commonly used as the first choice in children admitted [10]. Phenobarbital is an aromatic ASM thus in case of phenobarbital adverse allergic reactions, selection of alternative drugs that contain aromatic ring such as phenytoin and carbamazepine may produce severe reactions.

Levetiracetam is a relatively new non-aromatic ASM which is available on the market for treating adult and childhood seizures. Its antiseizure activity mechanisms are not still completely understood [11]. It is commonly used as monotherapy or adjunctive treatment of partial onset seizures with or without secondary generalization and adjunctive treatment for myoclonic seizures associated with juvenile myoclonic epilepsy and primary generalized tonic-clonic (GTC) seizures associated with idiopathic generalized epilepsy in adults [12]. Its other approved indications include as an adjunctive treatment of partial seizures, myoclonic seizures as well as of some epilepsy syndromes (i.e., Rolandic epilepsy and late-onset childhood occipital epilepsy) in children and infants in controlling focal and generalized epilepsy [13]. There are reports of cross-sensitivity to levetiracetam after using aromatic ASMs [[3], [9]], thus considering it as an alternative choice when hypersensitivity to another ASM has been happened is challenging. Due to the importance of this drug in controlling childhood seizure disorders and its increasing use in recent decade, the present study intends to investigate the cross-reactivity between phenobarbital and levetiracetam in children with epilepsy referring to two tertiary care hospitals in Tehran.

## Materials & methods

This study is a prospective, observational independent assessor study conducted over a period of three years (from September 2014 to the January 2018). The research population consists of all patients with different seizure types referring to Mofid children's hospital and Shohadaye Tajrish hospital (both are tertiary care hospitals located in Tehran) that have been treated with phenobarbital and had a hypersensitivity reaction to the drug.

The inclusion criteria were the age range between one month to 18 years, hypersensitivity reactions due to phenobarbital monotherapy treatment for seizure control, and that the hypersensitivity and allergic reactions were not because of another reason beside phenobarbital use. Phenobarbital doses prior to hypersensitivity reaction were not collected from the patients. This decision was based on the fact that due to previous studies hypersensitivity reactions to ASMs are not dose-dependent [[14], [15], [16]]. After hospitalization, the treatment for hypersensitivity was started, which included phenobarbital abrupt discontinuation and daily administration of 1 to 2 mg/kg of prednisolone for one week. At the first day of hospitalization and after phenobarbital discontinuation, benzodiazepine with daily dose up to 1 mg/kg (mean daily dose of 0.8 mg/kg) was used for seizure control until complete recovery from hypersensitivity reaction. The duration of benzodiazepine therapy varied between patients. It ranged from 1 to 3 weeks with the mean duration time of 12 days. At the end of benzodiazepine therapy, the dose of benzodiazepine was gradually reduced over 1 to 2 days until complete discontinuation, and simultaneously levetiracetam was introduced to the patients with the loading dose of 30 mg/kg and maintenance daily dose of 10 mg/kg. At the time of discharge, a dose of 20 to 60 mg/kg of levetiracetam (mean dose of 40 mg/kg) was administered daily. Patients were followed up for six months and visited monthly. Parents and caregivers were asked to report to the physicians in case of any signs of increased skin sensitivity. If there was any seizure recurrence in a patient, the patient would have gone under a second 6 months of follow up to control the seizure.

Demographic and clinical data of the patients were collected at the beginning of the research process and after the replacement of the drug by using a checklist. Any allergic reactions and any seizure recurrences were recorded and reported after the observation period was over. The data were analyzed using SPSS statistical software version 25.0 (IBM, Armonk, NY, USA). A significant level of 0.05 was considered.

Informed consent was taken from the parents of the children before enrolling and this study was conducted as per the Declaration of Helsinki and has been approved by the Ethics Committee of Shahid Beheshti University of Medical Sciences, Tehran, Iran (IR.SBMU. MSP.REC.1396.750).

## Results

In this study, 30 patients with phenobarbital hypersensitivity reactions were enrolled, 14 of them (47 %) were males and 16 (53 %) were females. The mean age of patients was 42.4 months. The lowest age at the time of the admission was 6 months and the highest age was 8 years old. 27 % of patients were children of consanguineous marriage. Of these, three children’s parents were first-degree relatives and five were second-degree families. In six (20 %) of patients, a history of allergy had been reported: 3 cases of food allergy, 2 cases of drug allergy and one history of respiratory allergy.

**Fig. 1:**
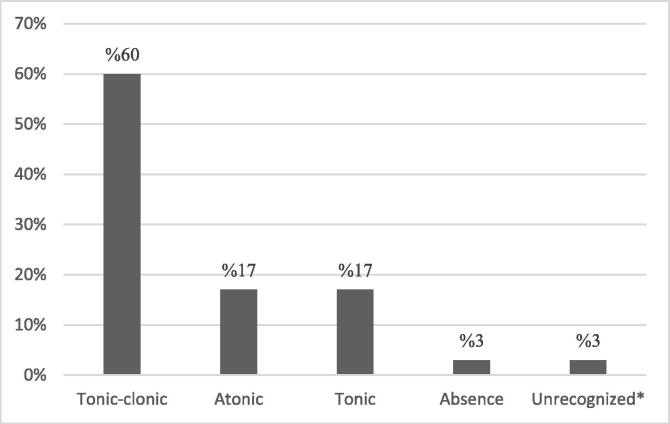
Distribution of patients by type of seizure. * The unrecognized group included seizures which could not be classified due to inadequate information or inability to place in other categories.

In 20 % of patients, there was a family history of seizure. 27 % of the patients had developmental disorder. In 30 % of the patients, there was a history of associated anomalies and in 7 % of patients, an infection was also diagnosed at the same time of drug allergic reactions occurrence. Participants in this study had different types of seizures which were categorized according to latest operational classification of seizure types by the international league against epilepsy [17], including Generalized onset seizures (classified as tonic-clonic, atonic, tonic, absence) and unrecognized seizures. The unrecognized group included seizures which could not be classified due to inadequate information or inability to place in other categories. Information about the seizure types of the patients is shown in Fig. 1.

In terms of laboratory criteria, 2 cases of leukocytosis and 3 cases of impaired liver tests have been observed. There was no case of eosinophilia in any of the patients. In terms of the type of response to phenobarbital in the examined patients, skin reactions in every 30 cases have been reported and 2 cases had mucosal skin reactions. None of the patients had respiratory and digestive problems.

Patients were followed up for six months after replacing phenobarbital with levetiracetam. Based on the collected data, no cross-reactive responses were observed in any of the patients under follow up.

In 70 % of patients, there were no seizures six months after administration of levetiracetam. Seizure recurrences reported in the rest of patients were managed with increasing dosage in the second six months of follow-up, which decreased seizure recurrence rate to 10 %. Comparison of demographic and clinical variables based on Seizure recurrence in 6 months follow up can be seen in Table 1.

**Table 1:** Demographic and Clinical Variables based on Seizure recurrence in 6 months follow up.

	Seizure recurrence Count = 9 cases	No seizure recurrence Count = 21 cases	P-Value
Average age	28.1 ± 44.9	25.1 ± 41.4	0.737
Male sex	6(66.7)	10(47.6)	0.440
Family history of seizures	4(44.4)	2(9.5)	0.049
developmental delay	6(66.7)	2(9.5)	0.003
History of associated anomalies	6(66.7)	1(4.8)	0.001
Children of consanguineous marriages	5(55.6)	3(14.3)	0.032

According to Table 1, there is a significant relationship between family history of seizure, developmental delay, history of associated anomalies and being children of consanguineous marriages with seizure recurrence (P < 0.05).

Investigations of 9 cases with recurrence of seizure indicate that 3 patients were neurotypical and 6 had developmental disorders. Of the six patients with developmental disorders, 3 cases had structural disorders, 1 had metabolic disorder and 2 were idiopathic. Three cases of the patients with developmental delays had atonic seizures and other patients with developmental disorders had generalized seizures. In patients with seizure recurrence, there was a family history of seizure in 4 of them and 5 were children of consanguineous marriages.

## Discussion

In recent years, side effects of ASMs in children have been rising [18]. Anticonvulsant medications are one of the most common causes of unwanted immunologic side effects (i.e., AHS, delayed reactions like morbilliform and bullous exanthems, fixed eruption syndrome, TEN, SJS, and the overlap of TEN/SJS) in children and among these drugs phenobarbital is the most commonly used [[19], [20]].

Selecting the right alternative in case of allergic reaction and hypersensitivity to ASMs is very important, because the wrong choice may lead to dangerous lethal reactions such as SJS, TEN, DRESS or AHS in the patient. In this regard, the present study attempts to determine the effect of levetiracetam as an alternative to phenobarbital and investigate the cross-reactivity of this drug in children with epilepsy referring to two tertiary care hospitals in Tehran. The incidence of allergic reaction recurrence to the alternative drug and the drug’s efficacy on controlling seizure of the participants were evaluated to determine if levetiracetam could be a suitable substitute for phenobarbital susceptibility.

In this study the sexual distribution of patients was balanced; 30 patients were examined which 16 (53 %) were female. This was based on the results of previous studies which reported no gender differences in the ASMs’ hypersensitivity occurrence including a study conducted by Wang et al. in which 3793 patients were evaluated for skin rashes in the setting of allergic reactions to ASMs. 137 patients reported allergic skin rashes which 73 (53.28 %) of them were females [4].

Genetic polymorphism plays an important role in body’s immunologic responses to drugs and their mediators; This means different persons immunologic systems can react differently to a certain drug. Hertl et al. revealed the role of T lymphocytes in the pathogenesis of drug-induced cutaneous reactions as the peripheral blood mononuclear cells from patients with drug-induced immediate-type or delayed hypersensitivity reactions can be stimulated in vitro with the causative agent [21]. Mechanisms involved in immune mediated drug allergies is not completely understood but there are two major models to describe its mechanism. One is the hapten model which assumes drugs, like haptens, bind to a cellular protein then together with the protein are processed and presented to the immune system. The other model is direct recognition model which assumes the binding of drugs to any protein is not necessary and *T*-cells receptor can recognize the drug together with the major histocompatibility complex (MHC)/peptide complex, causing drug allergy [22]. These models also explain the difference in immunologic responses between patients which is based on genetic differences.

The role of genetic polymorphism in cross-reactivity and its potential application was well established in previous studies for example Wang et al. proposed that the HLA-B*1502 allele may be associated with a pattern of cutaneous adverse drug reactions (cADRs) in response to cross-reactivity to ASMs. They recommended testing for the presence of the HLA-B*1502 for any patient with cross-reactivity to ASMs before introducing another ASM [23]. Their results were in agreement with another study conducted by Zhang et al. showing the significant association of HLA-B*1502 and HLA-B3101 in cADRs in Chinese population [24].

Considering the role of genes on immunologic reactions factors such as age, race, family and allergy history may have a significant influence on the drug sensitivity, its severity, and possible cross-reactions to other ASMs [25]. In the present study, 6 cases (20 %) of the patients had a history of allergy including 3 cases of food allergy, 2 cases of drug allergy and 1 respiratory allergy. Thus, studying these factors is recommended in subsequent studies to provide appropriate results for comparison.

In the present study, the drug allergic reactions of patients treated with levetiracetam, after allergic reaction to phenobarbital, were zero. This was consistent with the study of Wang et al., which reported that the incidence of hypersensitivity reactions with levetiracetam is less than 1 % [4].

This study’s aim was not to compare the efficacy of phenobarbital and levetiracetam but our findings indicate that after the replacement of phenobarbital with levetiracetam, only 30 % of children previously seizure free on phenobarbital had a recurrence of seizures on levetiracetam.

It should be noted that in the present study, 8 patients had neurodevelopmental disorders, 2 had metabolic problems and 5 had the structural problem of the nervous system and, according to the findings, there was a significant relationship between family history of seizure, developmental delay, history of associated anomalies, being children of consanguineous marriages and the likelihood of seizure recurrence in the follow-up period of six months which was in agreement with a review study by Rizvi et al [26] and a prospective cohort study conducted by Ramos-Lizana et al. [27].

Our prospective, observational study holds some limitations that are needed to be noticed. This study was conducted on a limited number of participants and institutions. The duration of follow-up was also limited. The cross-reactivity of other ASMs were not investigated and there was heterogeneity in seizure types of participants. Other potential risk factors for seizure recurrence besides family history of seizure, developmental delay, history of associated anomalies and being children of consanguineous marriages were not investigated. Further studies should be conducted to study these issues in different types of seizures with a larger sample size and wider range of people and ASMs considering other potential influencing factors which can alter the cross-reactivity response to these drugs.

## Conclusion

In conclusion, based on the findings of this study, in the case of a drug allergy to phenobarbital, levetiracetam may be a reasonable choice as an alternative because of the low level of seizure recurrence after taking the drug and low incidence of cross-sensitivity.

## Declaration of Competing Interest

The authors declare that they have no known competing financial interests or personal relationships that could have appeared to influence the work reported in this paper.
